# Management of type 1 immediate hypersensitivity reactions to antituberculosis drug: succesful desensitization

**DOI:** 10.1186/s13223-022-00737-4

**Published:** 2022-11-21

**Authors:** Zeynep Yegin Katran, İsmet Bulut, Aylin Babalik, Metin Keren

**Affiliations:** 1 Department of Allergy and Immunology, University of Health Sciences, Süreyyapaşa Training and Research Hospital, Istanbul, Turkey; 2 Department of Chest Diseases, University of Health Sciences, Süreyyapaşa Training and Research Hospital, Istanbul, Turkey

## Abstract

**Objective:**

In this study, it was aimed to investigate the prevalence of type 1 hypersensitivity reaction under tuberculosis treatment and the management of hypersensitivity.

**Methods:**

The study is a case series. All of the patients who were hospitalized between 01.02.2015–01.05.2021 were examined. All patients who developed a drug-induced type 1 immediate hypersensitivity reaction were included. Antituberculosis drugs were given with the protocol made by Buhari et al. However, unlike what is stated in the protocol, pyrazinamide was given last during the administration of the drugs.

**Results:**

2677 patients received inpatient tuberculosis treatment; type 1 immediate hypersensitivity reactions were seen in 94 (3.5%) patients. Due to missing data in the file, 81 patients were included in the study. 44 (54.3%) of the cases were women; mean age (mean ± SD) 50.7 ± 17.69 years; 76 (93.8%) of them are citizens of the Republic of Turkey; 58 (71.6%) of them were diagnosed bacteriologically; 65 (80.2%) of them were pulmonary tuberculosis. The most common skin finding was urticaria in 49 (60.5%). The drug responsible for the most common reaction was pyrazinamide. In 49 (60.5%) cases, drugs were given by desensitization and it was successful. The duration of treatment was 7.91 ± 2.5 months (6–18 months). When evaluated in terms of treatment results, 68 (84%) patients successfully completed the treatment.

**Conclusion:**

Our study is the largest series of patients who developed type 1 immediate hypersensitivity reaction while receiving antituberculosis treatment. A practical, easy desensitization scheme has been shared.

## Introduction

Tuberculosis is the leading cause of death from infectious disease in adults worldwide. In drug-sensitive tuberculosis patients, treatment is started with isoniazid, rifampicin, ethambutol and pyrazinamide [1, 2]. Drug hypersensitivity in tuberculosis is an important problem affecting the treatment process. All the antituberculosis drugs can be responsible for hypersensitivity reaction. Hypersensitivity reactions to antituberculosis drugs occur in %3–%4 of patients and also usually cutaneous and benign, seen in the first week of treatment. IgE mediated anaphylaxis to anti tuberculosis drugs are quite rare [3]. Rifampicin and pyrazinamide are the most common drugs caused hypersensitivity reactions [3–5]. Although treatment is initiated with isoniazid, rifampicin, ethambutol and pyrazinamide, drug changes may be required due to hypersensitivity. The presence of isoniazid and rifampicin in the regimen provides the advantage of shorter treatment time [6]. Management of hypersensitivity is very important due to long treatment time and increased side effects with secondary drugs [7]. Successful desensitization in hypersensitivity is very important in terms of patient compliance, treatment success and treatment cost. There are different desensitization protocols applied for early-type IgE-mediated reactions in tuberculosis [8–11]. Although different schemes are recommended for each drug, all patients were successfully desensitized with the desensitization scheme proposed by Buhari et al. [8, 10–13]. We shared the characteristics of our patients who developed IgE-mediated hypersensitivity while receiving tuberculosis treatment, our treatment management and the success of the desensitization scheme.

## Material-methods

The design of the study was a case series. 18 years and over; all inpatients who developed type 1 immediate hypersensitivity were included in the study between 01.02.2015 and 01.05.2021. Our hospital is a tertiary hospital and is located in Turkey's largest metropolis such as Istanbul. It is one of the 4 hospitals where patients with resistant tuberculosis are consulted. Therefore, the general agreement of our results may be limited. The primary aim of the study is to determine the appropriate treatment scheme in type 1 hypersensitivity reaction; The secondary aim is to determine the treatment management of type 1 hypersensitivity reaction.

All of the patients were inpatients and the hypersensitivity reactions that developed after drug treatment were confirmed by Allergy and Immunology specialists. Patients who did not receive desensitization in accordance with the scheme and patients with non-tuberculosis mycobacterial infections were not included.

Demographic data of the patients, diagnosis of tuberculosis, clinical features of type 1 immediate hypersensitivity reaction and time of occurrence, drug treatments, and treatment results were evaluated. Age, gender and nationality were noted in demographic data. According to the World Health Organization (WHO), countries were evaluated in 6 different regions according to their level of development. These were Africa, the Americas, the Eastern Mediterranean, Europe, Asia and the Western Pacific [14]. In our study, cases were also analyzed according to WHO country grouping.

Tuberculosis diagnoses, organ involvement and treatments were evaluated as determined in the Turkish Ministry of Health Tuberculosis Diagnosis and Treatment Guide published in 2019 [2]. Diagnoses were classified as smear positive, culture positive, molecular test positive, histopathological diagnosis, clinical radiological diagnosis; organ affected from tuberculosis were classified as pulmonary and extrapulmonary; the extrapulmonary group was grouped as miliary, lymph node, pleura, kidney, pericardium, larynx.

When the type 1 immediate drug hypersensitivity reaction was mentioned, sudden redness, itching, urticaria, angioedema, rhinitis, conjunctivitis, bronchospasm, abdominal pain, syncope, anaphylaxis, which develops in the first hour after taking the drug but could last up to six hours were evaluated. Type 1 immediate hypersensitivity reactions were (usually) IgE mediated, involve mast cell degranulation [15]. Desensitization was a method to get the tolerance [16, 17]. Patients who developed a hypersensitivity reaction were treated first. After all symptoms had resolved, the drugs were given by desensitization according to the specified scheme. The day when hypersensitivity developed, the success of desensitization, and the time to completion of tuberculosis treatment were evaluated.

In our clinic, before we had started this study, we had seen prick and intradermal test of drugs (isoniazid, ethambutol,rifampicin, pyrazinamide) were negative in 10 healthy volunteers. In our country, isoniazid tablet, ethambutol tablet, pyrazinamide tablet and rifampicin ampoule forms were available. Prick test was performed by crushing isoniazid, ethambutol, pyrazinamide tablets and diluting them with 2 cc saline. Prick test was performed without diluting with rifampicin ampoule. An intradermal test was performed with only rifampicin ampoule. Intradermal testing with rifampicin was performed at a concentration of 0.002 mg/mL [18]. If prick test and intradermal test were performed, it was available in the patient files. The files were also examined in this respect.

Drug doses in desensitization were made as suggested by Buhari et al. [10]. However, unlike what was stated in the protocol, pyrazinamide was given last during the administration of the drugs. Each drugs were given with at least 6 steps and 2.5 times increase in dose, at intervals not exceeding 30 min [4, 19]. Patients were not given any premedication (ie antihistamines, systemic steroids) before starting the desensitization. The drugs were given under the supervision of a physician and nurse in our outpatients clinic (All patients were hospitalized in the tuberculosis service and were consulted to our outpatients clinic). One drug was given every day. If hypersensitivity did not develop, the next day after the full dose of the previous drug was given, the new drug was added by desensitization. When hypersensitivity developed, first hypersensitivity treated according to international guidelines. Later drugs were given again by desensitization. After the regime was completed, the patient was given all drugs for several days; patient was discharged after it was seen that patient could take regime without any problems.

**Table:** 

Desensitization scheme applied in patients with type1 immediate hypersensitivity reaction:	
Day 1	08:00: Isoniazid Solution A: 1 tablet of 300 mg of isoniazid is diluted with 40 cc of 0.9% NaCl The resulting concentration is 7.5 mg/mL 08:00 1 cc solution A-7.5 mg 08:30 2 cc solution A-15 mg 09:00 3 cc solution A-22.5 mg 09:30 1/8 tablet of isoniazid-37.5 mg 10:00 ¼ tablet of isoniazid-75 mg 11:00 ½ tablet of isoniazid-150 mg
Day 2 08:00: 300 mg of isoniazid	09.00: For rifampicin Solution B: 2 capsules of 600 mg of rifampicin are diluted with 60 cc of 0.9% NaCl. The resulting concentration is 10 mg/ml 08:00 1 cc solution B-10 mg 08:30 2 cc B solution-20 mg 09:00 5 cc B solution-50 mg 09:30 10 cc B solution-100 mg 10:00 20 cc B solution-200 mg 11:00 22 cc B solution-220 mg
Day 3: 08:00: isoniazid 300 mg + rifampicin 600 mg	09.00: For ethambutol Solution C: 1 tablet of 500 mg ethambutol is diluted with 10 cc of 0.9% NaCl. The resulting concentration is 50 mg/ml Solution D: It is taken from 2 cc of C solution. It is diluted with 18 cc 0.9% NaCl. The resulting concentration is 5 mg/ml 08:00 1 cc D solution-5 mg 08:30 2 cc solution D-10 mg 09:00 4 cc D solution-20 mg 09:30 8 cc D solution-40 mg 10:00 2 cc solution C-100 mg 10:30 4 cc solution C-200 mg 11:00 One tablet ethambutol-500 mg 12:00 1 + ¼ tablet ethambutol-625 mg
Day 4 08:00: isoniazid 300 mg + rifampicin 600 mg + ethambutol 1500 mg	09.00: For pyrazinamide Solution E: 1 tablet of 500 mg pyrazinamide is diluted with 10 cc of 0.9% NaCl. The resulting concentration is 50 mg/ml F solution: It is taken from 3 cc E solution. It is diluted with 27 cc 0.9% NaCl. The resulting concentration is 5 mg/ml 08:00 2 cc solution F-10 mg 08:30 4 cc F solution-20 mg 09:00 8 cc F solution-40 mg 09:30 16 cc solution F-80 mg 10:00 3 cc E solution-150 mg 10:30 4 cc solution E-200 mg 11:00 One tablet of pyrazinamide-500 mg 12:00 2 tablets of pyrazinamide-1000 mg
Day 5: HRZE	
Day 6: HRZE	
Day 7: HRZE	

In the statistics of the study; All analyzes were performed using SPSS 22.0. Differences in the mean were evaluated with the Mann–Whitney U test. Relative risk, odd ratios, and 95% confidence intervals were calculated. Chi-square and logistic regression analysis were used for categorical parameters.

Ethics committee approval of the University of Health Sciences, Süreyyapaşa Chest Diseases and Thoracic Surgery Training and Research Hospital was obtained for this study. (dated 03.06.2021, protocol code: 116.2017.R-225).

## Results

2677 patients received inpatient tuberculosis treatment between 01.02.2015 and 01.05.2021.; type 1 immediate hypersensitivity reactions were seen in 94 (3.5%) patients. Due to missing data in the file,the study population consisted of 81 patients. 42 (51.8%) of the cases were women. Mean age (mean ± SD) 50.7 ± 17.69 years; 76 (93.8%) of them were citizens of the Republic of Turkey; all of Asian descent; 58 (71.6%) of them were diagnosed bacteriologically; 65 (80.2%) of them were pulmonary tuberculosis; 4 (4.9%) of them had previously received antituberculosis treatment (Table 1).

**Table 1:** Demographic and clinical characteristics of the patients

Variable			N (%)
Gender	Female		42 (%51.8)
	Male		39 (%48.2)
Age	(mean ± SD)		50.7 ± 17.69
Nationality	Turkey		76 (%93.8)
		Uzbekistan	1 (%1.2)
		Turkmenistan	1 (%1.2)
		Syria	2 (%2.5)
		Georgia	1 (%1.2)
WHO country classification		Asia	81 (%100)
Diagnosis		Sputum positive	39 (%48.1)
		Culture positive	19 (%23.5)
		Molecular test positive	2 (%2.5)
		Histopathological	17 (%21)
		Clinical- radiological	4 (%4.9)
Organ affected from tuberculosis	Pulmonary		65 (%80.2)
	Extrapulmonary	Lymph node	7 (%8.6)
		Pleura	8 (%9.9)
		Kidney	1 (%1.2)
Prior treatment	No		77 (%95.1)
	Yes		4 (%4.9)

The most common skin finding was urticaria in 49 (60.5%) cases. Pruritus was observed in 21 (25.9%) cases, angioedema in 7 (8.5%) cases, and urticaria and angioedema in 4 (5%) cases. Chest pain, abdominal pain, syncope, rhinitis, anaphylaxis were not observed in any case (Table 2). Hypersensitivity reaction development time (mean ± SD) was 5.28 ± 15.24 days.

**Table 2:** Clinical features of hypersensitivity developed with tuberculosis treatment

	Female N (%)	Male N (%)	Total N (%)
Pruritus	9 (10.2%)	12 (15.7%)	21 (25.9%)
Urticaria	30 (35.8%)	19 (24.7%)	49 (60.5%)
Angioedema	3 (3.7%)	4 (4.8%)	7 (8.5%)
Urticaria + Angioedema	2 (2.5%)	2 (2.5%)	4 (5%)

When hypersensitivity developed, discontinuation of tuberculosis treatment was sufficient in 26 (32.1%) patients; In 36 (44.4%) patients, treatment was discontinued and antihistamine was given; Antihistamine and oral/parenteral steroids were given together in 19 (23.5%) patients. Anaphylaxis requiring adrenaline and oxygen support was not observed in any patient.

Of the 81 patients who developed a hypersensitivity reaction, 38 responsible drugs could be identified (Table 3). While receiving tuberculosis treatment, the treatment was interrupted when hypersensitivity developed. After the hypersensitivity was treated, the drugs were re-administered one by one with desensitization. In this study, the responsible drug could not be determined in patients whose desensitization was completed without any problems. Since the drugs were given one by one with desensitization, if hypersensitivity developed when the drug was given, it was accepted as the responsible drug. The most responsible drug was pyrazinamide in 17 cases. Subsequently, rifampicin, isoniazid and ethambutol were found to be responsible. Among all patients, one more drug were determined as the responsible agent in 7 (8.6%) cases.

**Table 3:** Drugs responsible for hypersensitivity

Responsible drug	Female	Male	Total
Isoniazide	6	1	7
Rifampicine	8	–	8
Etambutol	2	1	4
Pyazinamide	10	7	17
Streptomycin	–	–	–
Pas	–	1	1
Protionamide	–	1	1
Linezolide	1	–	1
Total	27	11	38

Skin prick test and intradermal test results were seen in 9 cases files. Among these cases, the isoniazid prick test was positive in one case. Intradermal tests with rifampicin ampoule were negative. When we looked Table 4, Case 5: we could see; prick test with isoniazid was positive. Isoniazid desensitization is complete with no problem. Urticaria developed while giving rifampicin with desensitization. After urticaria being treated, desensitization was completed at the second time. The treatment processes of the patients who underwent skin prick test and intradermal tests were seen at Table 4.

**Table 4:** Characteristics of patients undergoing diagnostic tests

Case	Skin finding	First treatment	Prick test positive	Rifampicine IDT positive	Long time treatment	Hypersensitivity responsible drug
1	Angioedema	HRZE	No	No	HRZE	Could not be determined
2	Urticaria				Isoniazide Rifampicine Etambutol Levofloxacin	Pirazinamid
3	Urticaria				HRZE	Could not be determined
4	Urticaria				Rifampicine Streptomycin Pyazinamide Moxifloxacin Cyloserine	Isoniazide Etambutol
5	Urticaria		Isoniazide		HRZE	Rifampicine
6	Angioedema		No		HRZE	Isoniazide
7	Angioedema				HRZE	Could not be determined
8	Urticaria				HRZE	
9	Angioedema				HRZE	

Among all patients, 49 (60.5%) patients were diagnosed with drug-sensitive tuberculosis and could be restarted with isoniazid, rifampicin, ethambutol and pyrazinamide desensitization without any problems.

Although the patients had drug- sensitive tuberculosis; 7 cases were switched to an alternative scheme due to hepatotoxicity. Characteristics of patients who developed hepatotoxicity were seen at Table 5.

**Table 5:** Characteristics of patients who developed hepatotoxicity

Case	Age Gender	Reaction day	Skin sign	First treatment	Hypersensitivity developing treatment	Desensitization applied regime	Responsible agent
1	40, F	0	Pruritus	HRZE	Isoniazide Ethambutol Moxifloxacin Cyloserine Amikasin	Isoniazide Etambutol Moxifloxacin Cyloserine AmIkasIn	
2	73, M	1	Urticaria		Etambutol Moxifloxacin Cyloserine Streptomycin	Etambutol Moxifloxacin Cyloserine Streptomycin	
3	18, M	1	Pruritus		Isoniazide Rifampicine Etambutol Moxifloxacin	Isoniazide Rifampicine Etambutol Moxifloxacin	
4	41, F	0	Pruritus		Isoniazide Etambutol Pyazinamide Moxifloxacin Cyloserine	Isoniazide Etambutol Pyazinamide Moxifloxacin Cyloserine	
5	63, M	2	Pruritus		Isoniazide Etambutol Pyazinamide Cyloserine Protionamid PAS	Isoniazide Etambutol Moxifloxacin Linezolid	PAS Protionamid
6	37, M	1	Pruritus		Isoniazide Rifampicine Etambutol Moxifloxacin	Isoniazide Rifampicine Etambutol Moxifloxacin	
7	56, M	1	Pruritus		Isoniazide Rifampicine Moxifloxacin	Isoniazide Rifampicine Moxifloxacin	

Treatment was initiated with isoniazid, rifampicin, ethambutol and pyrazinamide in 25 patients. Although desensitization was applied twice, at least one drug was changed duration of desensitization because of developed hypersensitivity.

The duration of treatment was 7.91 ± 2.5 months (6–18 months). When we evaluated treatment results, 68 (84%) patients successfully completed the treatment; 1 (1.2%) patient was excluded from follow-up; 3 (3.7%) patients died during the treatment process, and 9 (11.1%) patients are still under treatment.

## Discussion

This study consists of the largest series of patients with drug-sensitive tuberculosis who developed type 1 immediate drug hypersensitivity while receiving treatment.

In addition, studies reporting immediate hypersensitivity due to antituberculosis drug were associated with female gender [7, 20, 21]; There were also studies stating that hypersensitivity reactions were not affected by gender [22]. In our study, there was no difference between male and female genders.

The most common skin finding was urticaria; In the literature, the most common skin finding in type 1 IgE-mediated hypersensitivity was stated as urticaria [19, 23].

Our hospital was one of the major centers for tuberculosis in Turkey and was located in Turkey's largest metropolis, such as Istanbul. All of our patients were citizens of the Republic of Turkey and Asian origin. Looking at the literature, female gender, Asian origin, advanced age and HIV infection were associated with adverse reactions in antituberculosis treatment [4].

We think, all drugs should be discontinued in patients who develop hypersensitivity. Hypersensitivity should be treated. All drugs should be given individually with desensitization. In our study, the drug most responsible for hypersensitivity was pyrazinamide. For this reason, we think that the last addition to desensitization would be more appropriate.

Considering 9 patients who underwent diagnostic testing, the prick test was positive in isoniazid in one patient. This patient developed urticaria while receiving isoniazid, rifampicin, ethambutol, pyrazinamide treatment with the diagnosis of drug-sensitive tuberculosis. Tuberculosis treatment was discontinued and urticaria was treated. Patient was able to tolerate isoniazid with desensitization without any problems. In the same patient, urticaria developed when rifampicin was first added to the treatment with desensitization, but all drugs could be started again with desensitization for the second time. In yet another patient, the prick test and intradermal test performed with drugs were found to be negative; When drugs were started with desensitization, urticaria developed with isoniazid in the first desensitization, and urticaria with ethambutol developed in the second desensitization, and isoniazid-ethambutol was removed from the regimen and an alternative regimen was started. Skin testing was not particularly useful in predicting culprit drug in the patients where skin testing data was available. İt is seen that there is a need for validation in diagnostic tests performed with antituberculosis drugs.

When the drugs were re-administered without skin tests, sometimes the responsible drugs could be found. Finding responsible drug and removing from treatment were compatible with better treatment outcomes [24]. Restarting the treatment with desensitization, not removing strong drugs such as isoniazid and rifampicin from the regimen provided a shorter treatment time and treatment success [1, 2]. The duration of treatment with secondary antituberculosis drugs without first-generation agents increased to at least 18 months [6]. It was a more effective method to give the primary drugs by desensitization instead of immediately replacing them with secondary drugs [25]. We also recommend that primary antituberculosis drugs be administered by desensitization and, if unsuccessful, regimen changes were made.

When hypersensitivity developed in tuberculosis, we were faced with two situations. On one side, we could not continue to drug due to the hypersensitivity. On the other hand, when we interrupted the treatment, it was necessary to start the treatment quickly because of the risk of drug resistance and tuberculosis reactivation [5]. Bermingham et al. suggested that several different pathways could be followed when reintroducing drugs in type 1 IgE-mediated reactions. One of them was that all drugs could be given with rapid drug desensitization and this decision should be made for the patient [3]. Adverse drug reactions related to antituberculosis treatment could cause treatment failure, morbidity and mortality. This situation could lead to more frequent hospitalizations and more examinations, resulting in increased economic outcomes [26]. We believe that the right management will bring success.

The index reactions in this series were not severe. This may be related to the small number of our patients or severe reactions may be rare. In this regard, prospective studies with larger patient series are needed.

When we look at the treatment results, 84% of the patients successfully completed the treatment after desensitization. No treatment failure was observed. Although there were different schemes recommended for desensitization [11–13], we could say that our scheme was usable when we looked at our treatment results.

Our study was the largest series of patients who developed type 1 immediate hypersensitivity reaction while receiving antituberculosis treatment. A practical, easy desensitization scheme had been shared. Stop all drugs and then resume them one-by-one via desensitization is the most practical approach. This method allows for first line agents to be used, minimizes treatment duration, especially in the setting that skin testing does not seem particularly helpful in identifying culprit drug.

## Data Availability

The authors have no conflicts of interest to declare.

## Abbreviations

WHO: World Health Organization
HRZE: Isoniazide + Rifampicine + Etambutol + Pirazinamid
PAS: Paraamino salicylic acid
